# Tens of images can suffice to train neural networks for malignant leukocyte detection

**DOI:** 10.1038/s41598-021-86995-5

**Published:** 2021-04-12

**Authors:** Jens P. E. Schouten, Christian Matek, Luuk F. P. Jacobs, Michèle C. Buck, Dragan Bošnački, Carsten Marr

**Affiliations:** 1 Department of Biomedical Engineering, Eindhoven University of Technology, Eindhoven, The Netherlands; 2 Institute of Computational Biology, Helmholtz Zentrum München-German Research Center for Environmental Health, Neuherberg, Germany; 3 Department of Internal Medicine III, University Hospital Munich, Ludwig-Maximilians-Universität München-Campus Großhadern, Munich, Germany; 4 Department of Medicine III, Technische Universität München, Klinikum rechts der Isar, Munich, Germany

**Keywords:** Cancer imaging, Haematological cancer, Biomedical engineering, Software, Image processing

## Abstract

Convolutional neural networks (CNNs) excel as powerful tools for biomedical image classification. It is commonly assumed that training CNNs requires large amounts of annotated data. This is a bottleneck in many medical applications where annotation relies on expert knowledge. Here, we analyze the binary classification performance of a CNN on two independent cytomorphology datasets as a function of training set size. Specifically, we train a sequential model to discriminate non-malignant leukocytes from blast cells, whose appearance in the peripheral blood is a hallmark of leukemia. We systematically vary training set size, finding that tens of training images suffice for a binary classification with an ROC-AUC over 90%. Saliency maps and layer-wise relevance propagation visualizations suggest that the network learns to increasingly focus on nuclear structures of leukocytes as the number of training images is increased. A low dimensional tSNE representation reveals that while the two classes are separated already for a few training images, the distinction between the classes becomes clearer when more training images are used. To evaluate the performance in a multi-class problem, we annotated single-cell images from a acute lymphoblastic leukemia dataset into six different hematopoietic classes. Multi-class prediction suggests that also here few single-cell images suffice if differences between morphological classes are large enough. The incorporation of deep learning algorithms into clinical practice has the potential to reduce variability and cost, democratize usage of expertise, and allow for early detection of disease onset and relapse. Our approach evaluates the performance of a deep learning based cytology classifier with respect to size and complexity of the training data and the classification task.

## Introduction

Acute lymphoblastic leukemia (ALL) is a malignant disease that arises from one or more genetic alterations or chromosomal abnormalities and affects the differentiation and proliferation of lymphoid precursor cells^1^. ALL represents 80% of childhood leukemias^2^ with a relatively high 5-year survival up to 90%^3^, and 20% of adult leukemias^2^ with a lower long-term survival between 30% and 50%^4^. As for many other hematological diseases, cytomorphological examination of blood smears by experts is among the initial steps of the diagnostic workup of ALL. Typically, the presence of a blast fraction of at least 20% in blood or bone marrow is required for the diagnosis of acute leukemias^5^. Classification of single-cell images however has proven hard to automatise, which makes it time-consuming and sensitive to intra- and inter-expert variability^6^. Automatically detecting and classifying single blood cell images would improve standardization, speed up the diagnostic process, and allow for a larger number of cells per individual to be examined, increasing the significance of statistical analyses and enabling the identification of small cell subpopulations.

Detecting cells with a malignant morphology can be treated as an image classification problem and addressed with convolutional neural networks (CNNs). Several problems from medical imaging have recently been shown to be amenable to analysis with CNNs, e.g. skin cancer classification^7^ or mutation prediction^8^. For leukemia diagnosis, CNNs have been used, e.g., to distinguish between cases with favourable and poor prognosis of chronic myeloid leukemia^9^, or to recognise blast cells in acute myeloid leukemia^10^. However, the dependence of CNN performance on the size of the training set is highly relevant for medical applications, since expert annotation is often expensive and time consuming, making generation of large high-quality datasets difficult^11,12^. Much previous work has focussed on the overall size of the data sets^13^, whereas we are interested in exploring the effect of training set sizes on the overall performance. Systematic training set variation has been previously analyzed for CT image classification^14^. Here, we study the impact of training dataset size on model performance for microscopic images.

First a small publicly available dataset containing 250 single-cell images of leukocytes of ALL patients and healthy individuals is used for CNN-based cell type classification. Both a binary and multiclass classifier are trained on this dataset and the binary classifier performance is evaluated with respect to the number of training images used. While increasing the size of training data in a systematic manner, we investigate the performance of our CNN and analyze the focus of the network as a function of the number of training images. To evaluate the robustness and generalizability of our results, an analogous analysis is performed with a much larger publicly available acute myeloid leukemia (AML) dataset containing more than 18,000 single-cell images, with very similar results.

## Materials and methods

### ALL dataset

**Figure 1:**
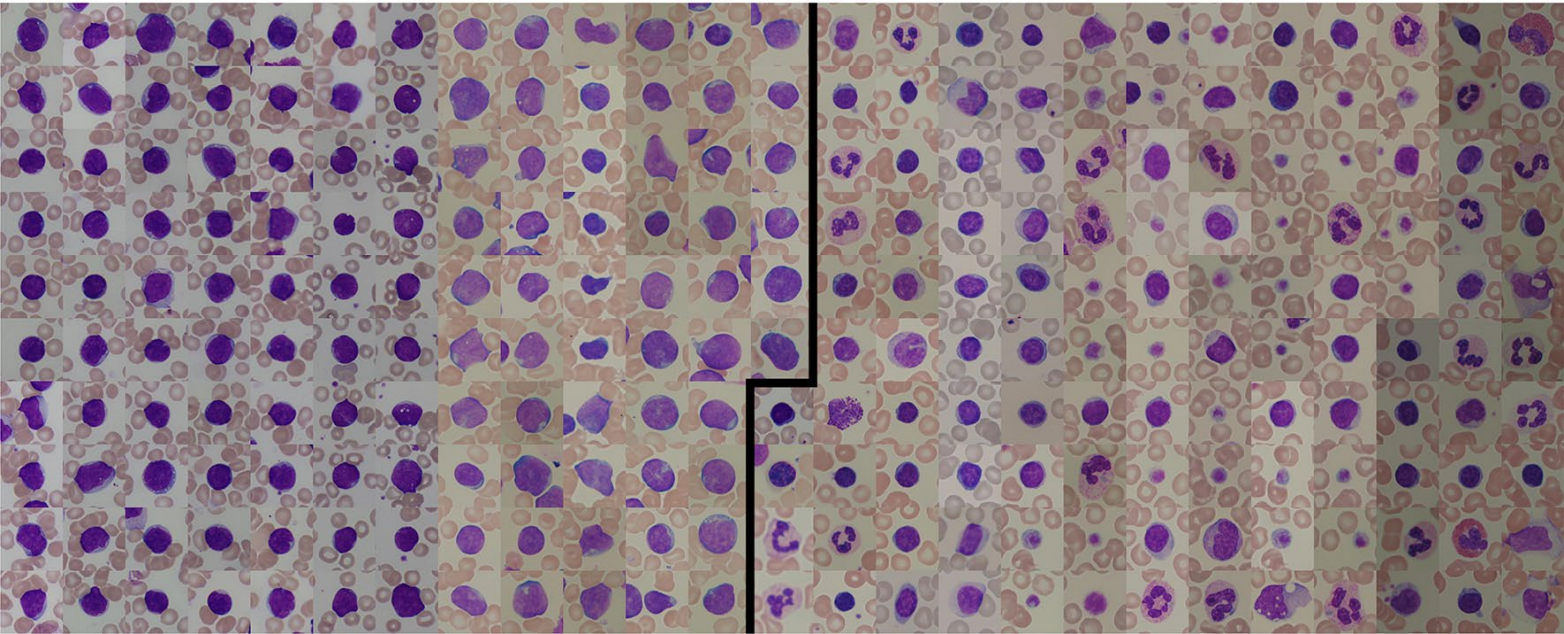
The ALL dataset of 250 single-cell images from Labati et al.^15^ used in this study. Blast cell images from ALL patients on the left are separated by a black line from thrombocytes and non-malignant leukocytes from healthy individuals on the right. Each image has a size of 257 × 257 pixels.

The ALL dataset used in this study contains 260 single-cell images and was obtained upon request from the Department of Information Technology at the University of Milan^15^. Ten images were identified as duplicates and removed. The original single-cell annotation is based on patient diagnosis (healthy vs. ALL patient). An overview of the full dataset is shown in Fig. 1. Half of the images are lymphoblasts from ALL patients, the other half are thrombocytes and other non-malignant types of leukocytes from healthy individuals. These single-cell images have been cropped from 108 larger photographs of the blood smear monolayer. Since multiple single-cell images could come from the same larger image and some of the larger images overlap, we assume that at least a subset comes from the same individual. However, detailed information on the number of subjects included and the number of single-cell images per subject is not available (personal communication with F. Scotti). Further patient information about subtypes or genetic alterations is not provided. All single-cell images have a size of 257 × 257 pixels.

To discriminate blood cell classes, we asked an expert cytologist to reannotate all single-cell images. The discrimination of the 250 images into ten different diagnostic cell types is shown in Table 1. The full re-annotated data is available as Supplementary Table S1.

**Table 1:** The re-annotated dataset.

Class	Number of images
Basophil	1
Erythroblast	1
Eosinophil	2
Lymphoblast	126
Lymphocyte (atypical)	12
Lymphocyte (typical)	52
Metamyelocyte	1
Monocyte	5
Neutrophil (segmented)	22
Thrombocyte	28
Total	250

An expert cytologist classified single-cell images into ten different types of blood cells. These expert labels are used for the multiclass classification and available as Supplementary Table S1.

### Binary classifier

**Figure 2:**
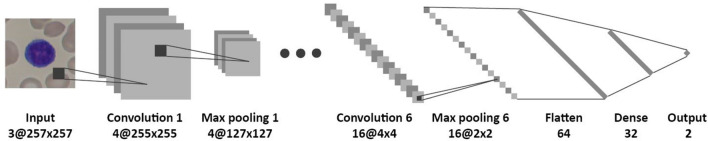
Network structure of the model used for binary classification. The network consists of six pairs of convolutional layers (with kernel size 3 × 3) and max-pooling layers. The feature maps are flattened and reduced to an output of size two. In total the network contains 7210 parameters.

To separate lymphoblasts from other leukocyte types, a sequential CNN^16^ with 7210 parameters (see Fig. 2) was trained with a binary cross-entropy loss function and softmax activation. The number of filters in the six convolutional layers are 4, 8, 8, 8, 16, 16. Tenfold cross-validation was used to assess classification performance. In each fold, 25 images (corresponding to 10% of the dataset) were left out to test the network after training. Of the 225 remaining images, 25 images were used for validation during training. We stopped training when the validation loss did not decrease for 50 epochs (“early stopping”). To evaluate how many single-cell images are required for an accurate classification, we systematically increased the size of the training data. Starting with ten training images, we each time added ten images (i.e., the old set is included in the new one) to the training set, trained a new network from scratch and evaluated on the same test set until the training set reaches the maximum 200 images. In the next fold, the 25 test images were again selected randomly from the set of images that have not been used for testing in previous folds. Thus, we ensure that every image is within the test set of precisely one fold. As no assignment of single-cell images to patients is provided in the dataset, a split into test and training set according to patient identity is not possible. However, patient-specific correlations between distinct single-cell images from the same blood smear have been shown to be insignificant^10^.

Data augmentation was used on the training set. Augmentation operations contained horizontal and vertical flipping and a random rotational transformation between 0° and 359°. This results in the addition of a random number of augmented images to the training set in each epoch. To evaluate the trained model we use the area under the curve of the receiver operating characteristic (ROC-AUC).

### Multiclass classifier

For multiclass prediction, the same network was used as in the binary case (see Fig. 2), but now with a categorical cross-entropy loss function, a softmax activation and a different output size which results in a CNN with 7342 parameters. We only included classes in the ALL dataset that contain five or more images (see Table 1) to ensure training-validation split, which results in six output classes. Again, tenfold cross-validation was used. Because the training set now contains multiple classes with different numbers of images, data augmentation was used to create 150 images of each class so that the training set was balanced. Because there is still an imbalance with respect to the number of images per class in the test set (which has not been augmented) we use the F1-score.

### AML dataset

CNN model training and evaluation was also applied to another, bigger AML data set from^17^, consisting of 18,365 images (400 × 400 pixels) with 15 morphological classes, which can be separated into 3294 blasts (myeloblasts and monoblast) and 15,071 non-blasts (the other 13 classes). The images used for training and validation were selected randomly from the dataset. Training of networks is done as described above, i.e., by incrementally adding images to the training set. Due to the large number of images available, testing is done in tenfold cross-validation using a balanced set of 600 unique images. For each fold the model is trained again with different random initializations. As above, we used early stopping when the validation loss did not decrease for 50 epochs. The only adaptation from the ALL to the AML dataset that had to be made in the neural network was changing the input shape from 257 × 257 to 400 × 400 pixels.

### Implementation

The deep learning model was implemented in Keras 2.0.8^16^ with Python 3.5 and Tensorflow-GPU 1.4.0 on an NVIDIA GeForce GT 730 GPU. Code is available via Github https://github.com/JensSchouten/ClassificationALL.

## Results

### A CNN distinguishes lymphoblasts from other cells with only 200 training images

**Figure 3:**
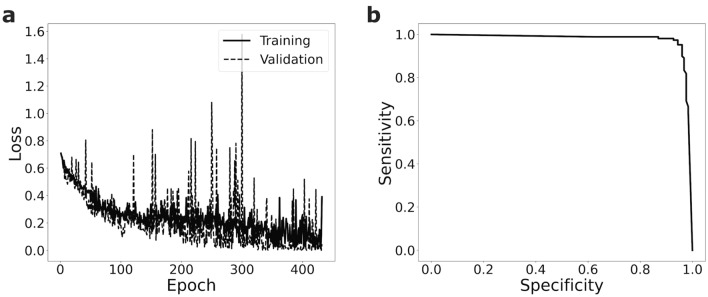
A CNN trained on the small ALL dataset is able to discriminate lymphoblast cells from other blood cells with high sensitivity and specificity. (**a**) During training one of the ten folds (training set size n = 200) the validation loss nicely follows the training loss. (**b**) The trained CNNs yield high receiver operating characteristics with an ROC-AUC of 0.97 ± 0.02 (mean ± s.d., n = 10 folds). Shown is the mean curve of the 10 folds.

To evaluate the impact of the training set size for recognizing malignant cells in blood smears, we train a sequential CNN (see “Methods” for details) for the binary classification task of discriminating lymphoblasts from all other cell types in the ALL dataset (see “Methods” and Fig. 1). We first use 200 images for training, 25 images for validation and 25 independent images for testing the classification performance. To evaluate the variability of the model, we use tenfold cross-validation (see “Methods”). For one of the folds, the training and validation loss is shown in Fig. 3a. In each fold, we use the validation set to select a model (see “Methods”). We then calculate sensitivity and specificity of our approach as a function of the chosen classification threshold. Averaging over the 10 folds, we achieve a high ROC-AUC of 0.97 ± 0.02 (see Fig. 3b and “Methods”).

### Already 30 images suffice for a good binary classification

**Figure 4:**
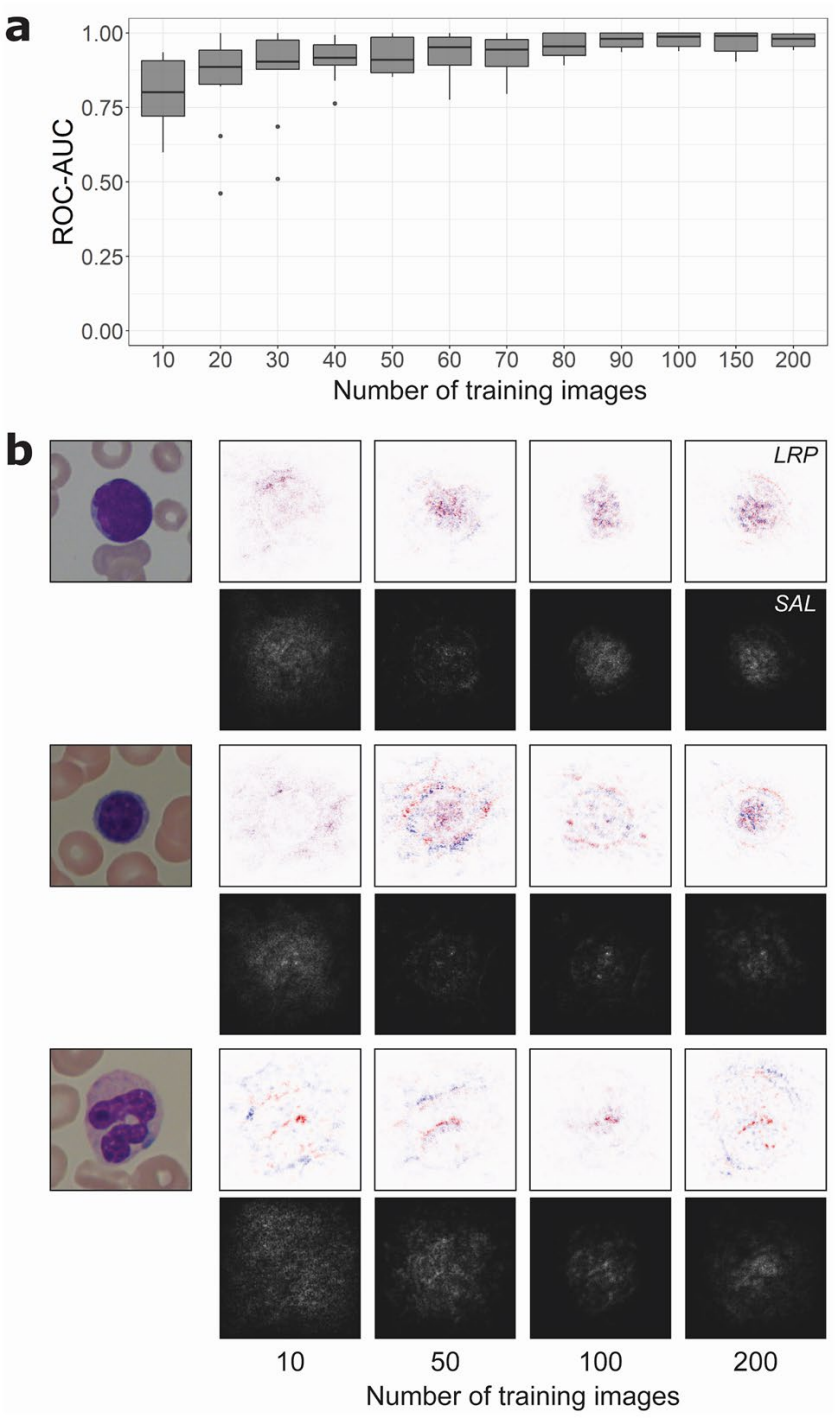
Lymphoblast classification performance and analysis as a function of training images from the ALL dataset. (**a**) Starting with ten training images, in each step ten images are added to the training set and the network is trained from scratch. The ROC-AUC of this network is calculated, and the process is repeated ten times so that all 250 images are at some point used as a training and validation set. Boxes show median and 2nd and 3rd quartile, with whiskers indicating the highest and lowest point within 1.5 times the interquartile range. Data points outside this range are considered outliers and visualized separately as dots. (**b**) Visualization with saliency maps (SAL) and layer-wise relevance propagation (LRP) when testing a lymphoblast (top), typical lymphocyte (middle) and segmented neutrophil (bottom) in networks trained on 10, 50, 100 and 200 images. In LRP, pixels visualized in red contribute positively to the final prediction, while pixels in blue carry negative relevance. In saliency maps, brighter pixels indicate a greater contribution to the final classification. Networks trained on 10 and 50 images seem to focus more on the surroundings of the leukocyte, while the networks trained on more images increasingly focus on the target cell itself and its nuclear structure.

We next systematically increase the training set size (see “Methods”), starting form ten images. We find that as expected the mean ROC-AUC increases with the number of training images, and that the variance decreases (Fig. 4a). However, after a relatively strong increase at the beginning, the ROC-AUC saturates at 30 images and increases only slowly when more images are added (see Fig. 4a and Supplementary Table S2).

To visualize which parts of the single-cell image a trained network focuses for classification, saliency maps^18^ and layer-wise relevance propagation (LRP)^19^ is used. Saliency maps show the relevance of individual pixels. In LRP, individual pixels that support the final classification of the network are colored red, whereas pixels speaking against the classification are colored in blue. Both methods are applied on networks with increasing training set sizes to study the difference in focus of these networks (Fig. 4b). For networks trained on ten single-cell images, relevant pixels are distributed within and around the leukocyte. With an increased training set of 50 images, regions in the cell nucleus gain more weight, a change that becomes more pronounced when the network is trained on 100 or 200 cell images. This may indicate that the network learns to focus on relevant regions of the image as it is trained on a larger dataset. Interestingly, the ROC-AUCs of training with 100 or 200 cell images are very similar (Fig. 4a).

**Figure 5:**
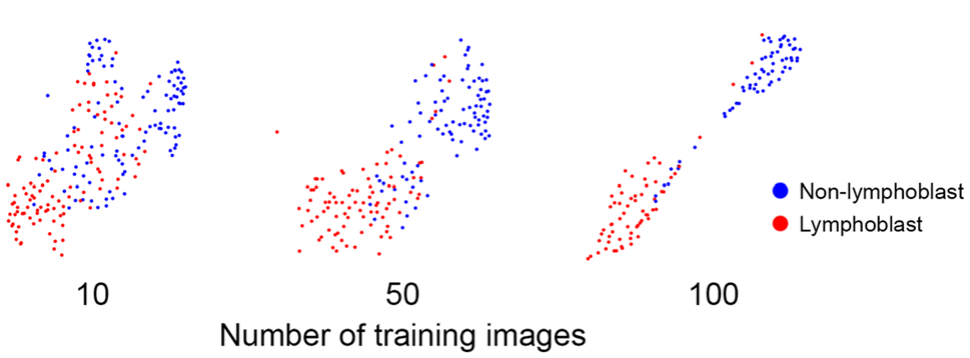
Separation of binary classes increases with increasing number of training images. tSNE reduces the 32 dimensions of the last hidden layer of networks trained with 10, 50, and 100 images to two dimensions. Coloring the last hidden layer for the respective 215, 175 and 125 lymphoblast and non-lymphoblast images shows an increasing cluster separation of the two classes with increasing training set size even though the ROC-AUCs for 50 or 100 training images are similar (see Fig. 2A).

To further study the internal representation of the network, we use tSNE^20^ for reducing the 32 dimensions of the last hidden layer to 2 dimensions. Figure 5 shows tSNE plots of networks trained with 10, 50 and 100 images from the same fold where the test set contains the original 25 images plus the images that were removed from the training set, which makes in total 215, 175 and 125 images. Visible is the increasing separation of the two classes, even between training sets with 50 and 100 images.

**Figure 6:**
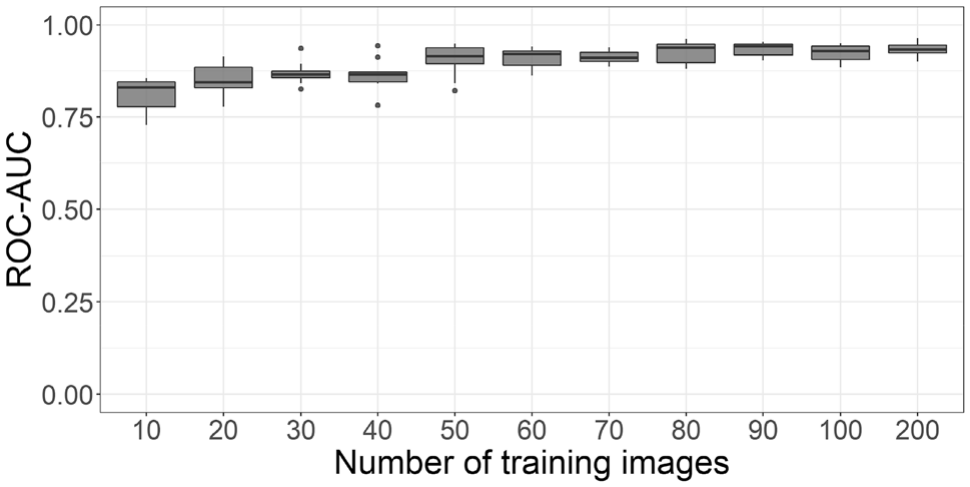
Blast cell classification performance as a function of training images for the AML dataset. We observe a saturation at around 50 training images. Boxes show median and 2nd and 3rd quartile, with whiskers indicating the highest and lowest point within 1.5 times the interquartile range.

To challenge our finding with a second, unrelated dataset, we repeated the analysis with a recently published data of over 18,000 single-cell images from the peripheral blood of AML patients (for access to the full annotated dataset, see^21^) and controls. Again, we increase the training set size by successively adding images (see “Methods”) to discriminate blasts from benign blood cells. As for the ALL data, the results suggest a saturation of performance already with a training set size of 30–50 of images (see Fig. 6). Using 50 training images results in an ROC-AUC of 0.91 ± 0.04 (mean ± standard deviation, tenfold cross validation) which increases to 0.93 ± 0.02 with 200 training images.

### Multiclass prediction

**Figure 7:**
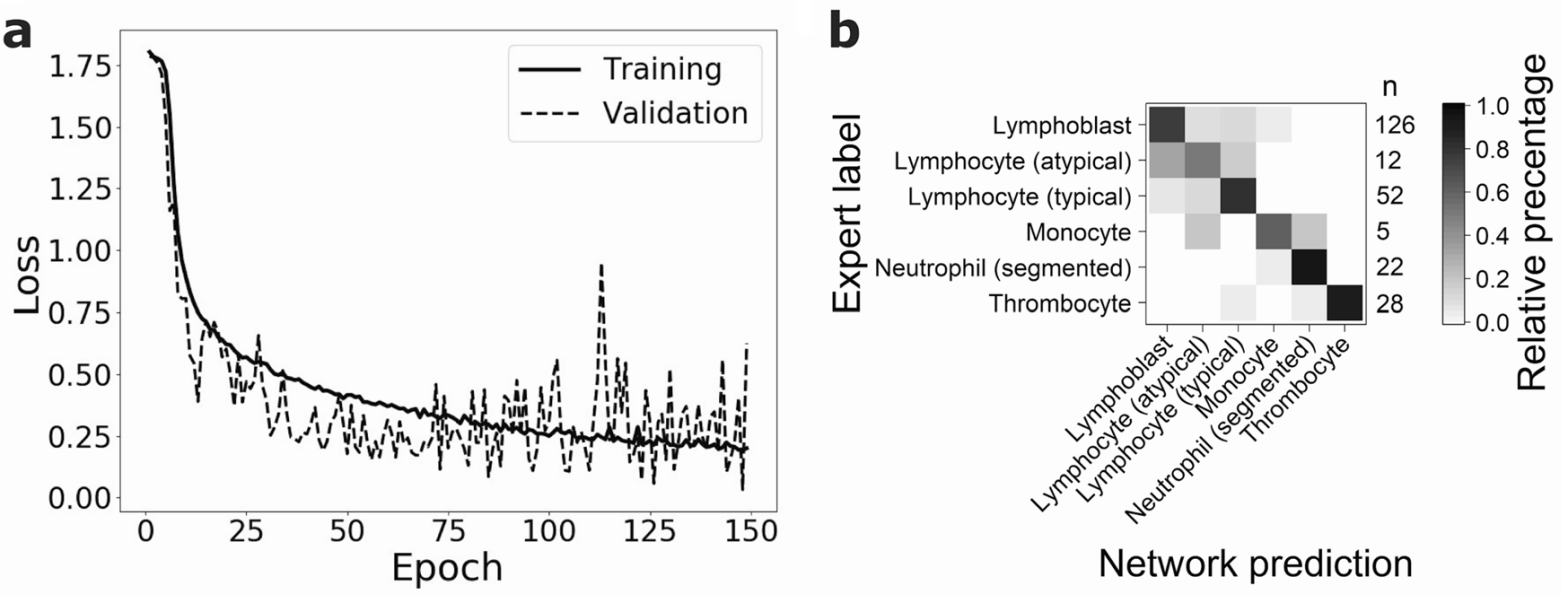
Multiclass classification of the ALL dataset using 200 training images. (**a**) Decrease of validation and training loss during training of one of the ten folds. Classification of the test set results in an F1-score of 0.81 ± 0.09 (mean ± s.d., n = 10 folds). (**b**) Confusion matrix visualizing the percentage of network predictions in each class. On the right hand side of the matrix the number of images in each class is given. In each class, 50% (in the case of atypical lymphocytes) or more cell images are classified correctly. Atypical lymphocytes (n = 12) and monocytes (n = 5) have the lowest number of images and show the most misclassifications.

In addition to the binary task, we also evaluated the classification performance of the same network for the classification of leukocytes in the small ALL dataset into six morphological classes (see “Methods”). Training (see Fig. 7a) and testing networks using tenfold cross-validation results in a F1-score of 0.81 ± 0.09. In each class, the majority of the images are classified correctly (see Fig. 7b). The largest number of misclassifications are visible for lymphoblasts, typical and atypical lymphocytes. As expected, classes with the lowest number of images (monocytes contain five images and atypical lymphocytes 12 images in total) show the poorest performance. When interpreting the multiclass predictions, it should be kept in mind that some morphological classes exhibit significant similarities and may be difficult to differentiate also for human examiners, who provide the ground truth used for training and evaluating the algorithms. Specifically, the typical and atypical lymphocyte classes may be difficult to discern, which is why a mixup between them has been considered tolerable (cf.^22^).

## Discussion

In this study we trained and evaluated a sequential CNN using a dataset of 250 images of blood cells from patients diagnosed with ALL and healthy controls. The network can distinguish lymphoblasts from normal leukocytes with an ROC-AUC of 0.97 ± 0.02. When varying the size of the training set, we find that increasing the number of training images beyond 30 only slightly increases the ROC-AUC of the binary classifier. However, saliency maps and LRP indicate that with increasing training set size the focus on nuclear features becomes more important. Similarly, tSNE embeddings of the final hidden layer suggest a clearer separation of lymphoblasts and non-lymphoblasts with an increasing amount of training data.

The ALL dataset from Labati et al.^15^ has been used repeatedly for automatic discrimination of blast versus normal lymphocytes. Using classical object detection, feature detection and machine learning classification, Joshi et al. achieved an accuracy of 93% with a kNN classifier, while Basima and Panicker^23^ achieved 94.6% with a support vector machine (SVM). Another study used the ALL dataset together with other datasets to perform a binary classification of blast and healthy cells and achieved an accuracy of 99.2% with a pre-trained ImageNet CNN^24^. This accuracy is only slightly higher than the performance of our network which is trained only on the ALL dataset. Multiclass classification was performed by two other studies where a CNN together with a SVM has been applied^25^ and achieved an accuracy of 99%, and a pre-trained CNN^26^ achieving 96.3%. In both cases a direct comparison with our results is difficult since in the training data of^11^ was supplemented by various single-cell images, and the added data is not publicly available. To evaluate the applicability of these methods for clinical purposes, a thorough comparison to the state of the art expert annotation would have to be performed.

We note that our network was both trained and tested on images from the same, limited dataset of 250 blood cell images. As little information is available on the statistical properties of this dataset, it is difficult to assess its homogeneity and representativity, or counter possible batch effects^27^. Moreover, the small size of the dataset may lead to significant sampling noise when splitting it for testing and training. Hence, we used an independent, much larger dataset to test if our findings are generalizable. This dataset consists of single-cell images from 200 individuals including 100 AML patients, and might therefore be expected to better represent variabilities in cell morphology and sample preparation. We followed the analogous approach as for the ALL dataset, and trained a binary classifier for myeloblast and monoblast recognition, using the identical network architecture. As for the smaller ALL dataset, we observed that classifier quality measured by the ROC-AUC increased with the training dataset size. Saturation of the ROC-AUC value was observed for a training set size of 50 images, only slightly more than for the much smaller ALL dataset. Hence, a high-quality binary classifier for recognition of one particular cell class of interest, i.e. lymphoblasts in the ALL dataset or myeloblasts and monoblasts in the AML dataset, can be obtained using a relatively small number of training images, on the order of tens of images. This is consistent with the observation that not all training samples of large datasets are equally important for training a network, which has motivated the development of importance sampling as a strategy to save computational resources in CNN development^28^.

Modifying the network such as to perform multiclass classification of blood cell images into six morphological categories resulted in an F1-score of 0.81 ± 0.09, indicating good performance. In the multiclass prediction task, misclassifications mainly occur for images taken from classes containing very few training samples, as well as for cell types that are known to be difficult to accurately discriminate morphologically, such as lymphoblasts, typical lymphocytes and atypical lymphocytes. Hence, as might be expected, also the multiclass classification performance depends on the number of training images and the complexity of the discrimination problem, i.e. the morphological similarity of target classes.

By systematically varying the size of our training set, we have shown that a relatively small number of training samples may be sufficient for training a network with good performance in both classification tasks. However, increasing the training set improves class separation, as indicated by gradient saliency maps, LRP, and low-dimensional embedding of the penultimate network layer.

Deep learning approaches have shown promise in several areas of medical image classification, in some cases attaining human-level performances using very large training sets. In clinical practice even a small prediction performance gain can be important. Consequently, large training sets should be used to full extent whenever available. However, this is not always the case, e.g., because their acquisition or annotation is not feasible, too time consuming or cost inefficient. In all such cases when one is confined to smaller datasets, our analysis is particularly relevant. As shown in this work, good results may already be obtained using a limited number of training images.

## Supplementary Information

Supplementary Table S1.

Supplementary Table S2.
